# Association of medical visit frequency with cardiovascular events and mortality in patients with dyslipidaemia: a cohort study using a Japanese claims database

**DOI:** 10.1186/s41043-026-01408-5

**Published:** 2026-08-02

**Authors:** Taku Matsunaga, Eiji Nakatani, Yasuto Sato, Hideaki Kaneda, Hiromitsu Ohno, Akira Sugawara

**Affiliations:** 1 Graduate School of Public Health, Shizuoka Graduate University of Public Health, 4-27-2 Kitaando, Aoi-ku, Shizuoka, 420-0881 Japan; 2 Department of General Medicine, Fujieda Municipal General Hospital, Shizuoka, Japan; 3 Laboratory of Biostatistics, NHO Nagoya Medical Center, 4-1-1 Sannomaru, Naka-ku, Nagoya, 460-0001 Japan; 4 Center for Health Informatics Policy, National Institute of Public Health, Saitama, Japan; 5 Okinaka Memorial Institute for Medical Research, Tokyo, Japan; 6 Allied Medical K.K, Tokyo, Japan

**Keywords:** Dyslipidaemias, Primary health care, Patient follow-up, Cardiovascular diseases, Stroke, Mortality

## Abstract

**Background:**

Dyslipidaemia is a major risk factor for cardiovascular and cerebrovascular diseases; however, the optimal frequency of medical visits remains unclear.

**Objective:**

To examine whether medical visit frequency is associated with the incidence of cardiac events, cerebrovascular events, and mortality among patients initiating lipid-lowering therapy.

**Methods:**

We conducted a retrospective cohort study using data from the Shizuoka Kokuho Database between April 1, 2012, and September 30, 2021. Patients initiating lipid-lowering therapy were classified into high-frequency (≥ 22 visits in 2 years) and low-frequency (8–12 visits in 2 years) groups. After 1:3 propensity score matching and exclusion of patients with events within 2 years, 1,435 patients in the low-frequency group and 4,412 in the high-frequency group were analysed.

**Results:**

Over a mean follow-up of 5.3 years, composite outcomes occurred in 5.6% of both groups (hazard ratio [HR] 1.08; 95% confidence interval [CI] 0.84–1.38; *p* = 0.571). No significant differences were observed for cardiac events (HR 1.13; 95% CI 0.83–1.54), cerebrovascular events (HR 1.13; 95% CI 0.67–1.90), or mortality (HR 0.94; 95% CI 0.60–1.48).

**Conclusion:**

Less frequent visits were not associated with increased risks of cardiovascular events or mortality. Extending visit intervals may therefore be a reasonable option for selected stable patients receiving lipid-lowering therapy for primary prevention.

**Supplementary Information:**

The online version contains supplementary material available at https://doi.org/10.1186/s41043-026-01408-5.

## Background

Dyslipidaemia, characterized by abnormal blood lipid levels, is a major risk factor for cardiovascular and cerebrovascular diseases [1–4]. In 2021, cardiovascular disease was the leading cause of death worldwide, with stroke ranking third; in Japan, these conditions ranked second and fourth, respectively, underscoring the importance of lipid management [5, 6]. Management of dyslipidaemia relies on lifestyle modification and pharmacotherapy. Regular clinical follow-up may influence treatment adherence and timely adjustment of therapy, and previous studies have suggested that consistent follow-up is associated with improved medication adherence and prevention of acute cardiovascular events [7, 8].

However, evidence regarding the optimal interval between visits remains limited. Japan is characterized by a high frequency of medical consultations, with the annual number of visits exceeding the OECD median (11–13 vs. 6.8 visits) [9]. In addition, the average prescription duration for dyslipidaemia is 38.1 days, and monthly visits are common [10]. Although such frequent visits may allow closer monitoring and treatment adjustment, it remains unclear whether they reduce major cardiovascular or cerebrovascular events or all-cause mortality.

As healthcare expenditures continue to rise in Japan, refillable prescriptions were introduced in 2022 as one policy response [11]. However, concerns remain that extending visit intervals could reduce preventive effectiveness, particularly given the debated cost-effectiveness of dyslipidaemia interventions [12]. Clarifying the association between visit frequency and clinical outcomes is therefore important from both clinical and health economic perspectives. This study examined whether visit frequency and interval affect the long-term incidence of cardiac events, cerebrovascular events, and mortality, specifically testing whether more frequent follow-up is associated with better outcomes.

## Methods

### Study design and data source

We conducted a retrospective cohort study using a new-user design [13], and data from the Shizuoka Kokuho Database (SKDB) between April 1, 2012, and September 30, 2021 ^14^. The SKDB contains detailed medical and long-term care insurance data for more than 2.5 million residents of Shizuoka Prefecture [14, 15]. We accessed the data between October 2023 and December 2024 and had no access to personally identifiable information during or after data collection. Japan has a universal health insurance system. The SKDB primarily includes beneficiaries of the municipal National Health Insurance system, which mainly covers self-employed individuals, unemployed individuals, and others not enrolled in employment-based health insurance, as well as beneficiaries of the Late-stage Elderly Medical Care System for adults aged 75 years or older.

### Population

**Fig. 1:**
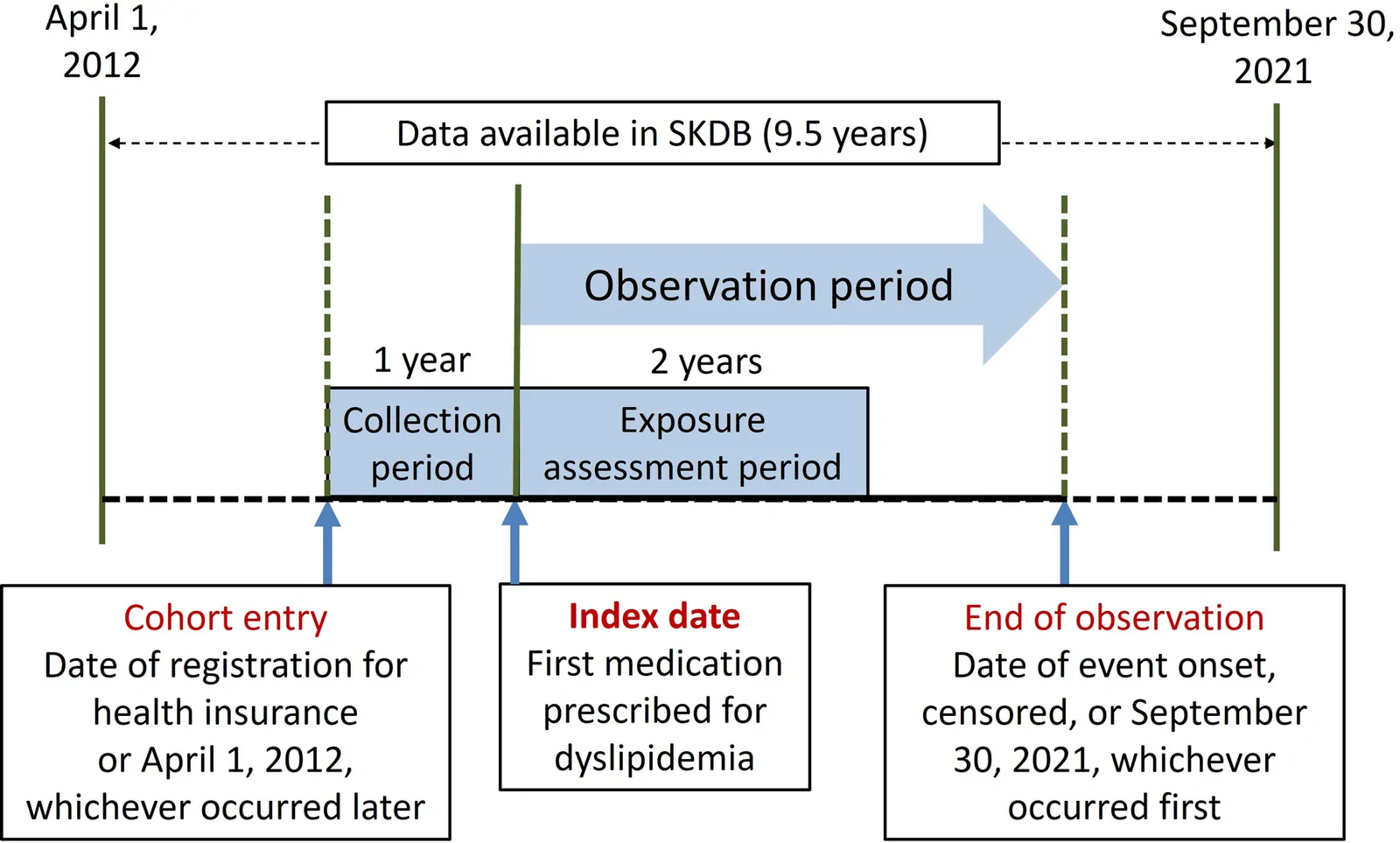
Study design for patients with newly treated dyslipidaemia in the Shizuoka Kokuho Database, Japan (2012–2021). SKDB, Shizuoka Kokuho Database

The study design is shown in Fig. 1. Patients were eligible if they had a new prescription for dyslipidaemia after a one-year baseline health evaluation and had undergone a health checkup within 6 months before treatment initiation. Dyslipidaemia-related diagnoses are listed in Supplementary Table S1. Dyslipidaemia medications were defined as agents classified under ATC code C10 (Supplementary Table S2). Patients with a history of cardiac or cerebrovascular events or cancer, those receiving maintenance dialysis, those self-injecting insulin, and those younger than 40 years were excluded.

### Group allocation according to frequency of medical visits

The index date was defined as the date of the first prescription for dyslipidaemia medication. Based on visit frequency during the 2 years after the index date, patients were classified into high- and low-frequency visit groups. The high-frequency group comprised patients who made ≥ 22 visits over 2 years, roughly corresponding to monthly visits, whereas the low-frequency group comprised those who made 8–12 visits, corresponding to approximately one visit every 2–3 months. Because medical reimbursement claims and chronic disease management fees in Japan are calculated on a monthly basis, this comparison was considered clinically meaningful. The low-frequency group was assumed not to include consecutive monthly visits. Patients who made 13–21 visits over 2 years were classified as an intermediate-frequency group and excluded from the primary analysis, but were included in the sensitivity analysis for comparison with the low- and high-frequency groups.

Visit frequency was determined from prescription records for dyslipidaemia medication. Patients with fewer than two visits during any 6-month interval (0–5, 6–11, 12–17, or 18–23 months) were considered not to have continued treatment and were excluded. The mean follow-up duration was 5.3 years.

### Outcome and potential confounders

The outcome was the interval from the index date to the occurrence of a composite of cardiac events, cerebrovascular events, and death; the corresponding codes are listed in Supplementary Table S3. Events occurring within 2 years of the index date were excluded because medication exposure and visit frequency were considered highly unlikely to influence them.

Potential confounders included age, sex, and comorbidities, including hypertension, diabetes, chronic kidney disease, dementia, rheumatic disease, liver disease, thyroid disease, and depression; the corresponding codes are listed in Supplementary Table S4. Smoking status, alcohol consumption, and the type of newly prescribed dyslipidaemia medication (statins, fibrates, and others) were also included. Clinical and laboratory variables included body mass index, systolic blood pressure, low-density lipoprotein cholesterol (LDL-C), triglycerides, high-density lipoprotein cholesterol, glycated haemoglobin, creatinine, aspartate aminotransferase, alanine aminotransferase, uric acid, and haemoglobin. In Japan, the Hisayama score is often used to assess atherosclerotic cardiovascular disease (ASCVD) risk in patients with dyslipidaemia [16, 17]. This score was used to classify patients aged ≤ 79 years into low-, medium-, and high-risk groups and to adjust for confounding.

Because LDL-C control may influence visit frequency, we also examined LDL-C levels within 1 year after treatment initiation.

### Statistical analysis

Summary statistics are presented for each cohort, with continuous variables expressed as mean ± standard deviation and categorical variables as number and percentage. Propensity scores were estimated using a multivariable logistic regression model incorporating the aforementioned potential confounders, excluding covariates with insufficient predictive contribution. The analysis cohort was constructed using 1-to-k propensity score matching with a caliper width of 0.2, where *k* did not exceed 10. Group balance was assessed using standardized mean differences, with values < 10% considered acceptable. Cumulative incidence curves were constructed. Between-group differences were evaluated using the log-rank test for survival and composite outcomes, and Gray’s test for other outcomes with death treated as a competing risk [18]. Point estimates and 95% confidence intervals (CIs) for cumulative incidence were calculated, and hazard ratios (HRs) with corresponding 95% CIs were estimated using Cox regression models.

To assess robustness, three sensitivity analyses were performed. First, groups were redefined according to the number of visits during the second year (months 13–24), with the high-frequency group comprising patients with 11–12 visits and the low-frequency group those with 4–6 visits. Second, the low-frequency group was expanded from 8 to 12 to 8–16 visits over 2 years, corresponding to one visit every 1.5-3 months, and compared with the high-frequency group comprising patients with ≥ 22 visits. Third, an intermediate-frequency group defined by 13–21 visits over 2 years was compared with both the low-frequency group (8–12 visits) and the high-frequency group (≥ 22 visits).

Subgroup analyses were conducted according to sex (female/male), age group (40–59, 60–69, 70–79, and ≥ 80 years), hypertension (yes/no), diabetes (yes/no), ASCVD risk (low, medium, or high), and type of lipid-lowering medication (statin or other). Differences between the low-frequency and high-frequency groups across subgroups were assessed using interaction terms in Cox multivariable regression models. A two-sided p-value < 0.05 was considered statistically significant, except in subgroup analyses, for which a p-value for interaction < 0.1 was considered significant. All analyses were performed using EZR Version 1.61 (Saitama Medical Center, Jichi Medical University, Tochigi, Japan) [19] and SAS Version 9.4 (SAS Institute Inc., Cary, NC, USA).

### Ethics statement

The study was approved by the ethics committee of the Graduate School of Public Health, Shizuoka Graduate University of Public Health, Japan (SGUPH_2021_001_074 − 2) and conducted in accordance with the Declaration of Helsinki. Informed consent was waived because the SKDB data were anonymized [20].

## Results

### Study population and patient characteristics

**Fig. 2:**
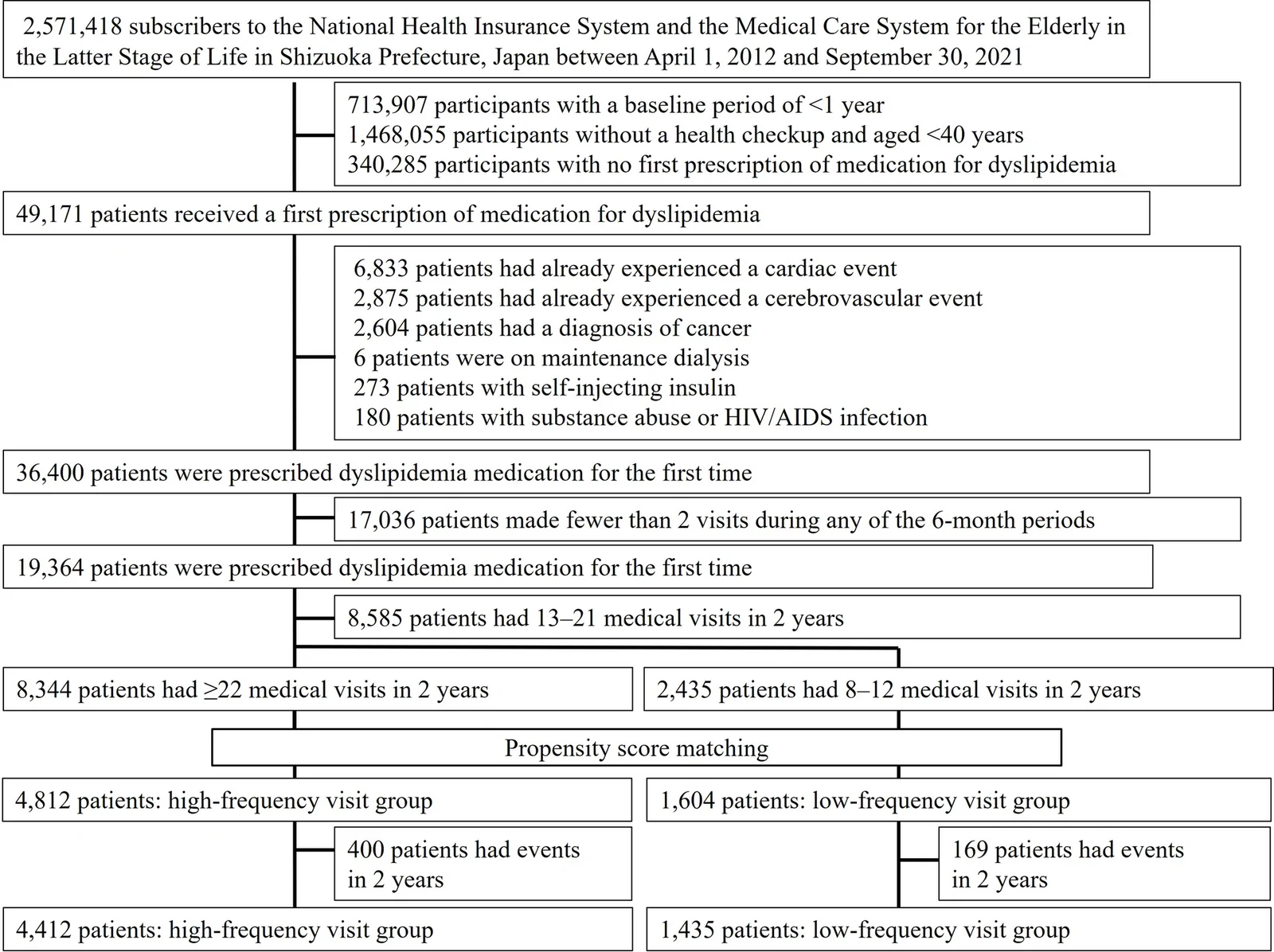
Flow chart of study population selection from the Shizuoka Kokuho Database, Japan (2012–2021). AIDS, acquired immunodeficiency syndrome; HIV, human immunodeficiency virus

**Fig. 3:**
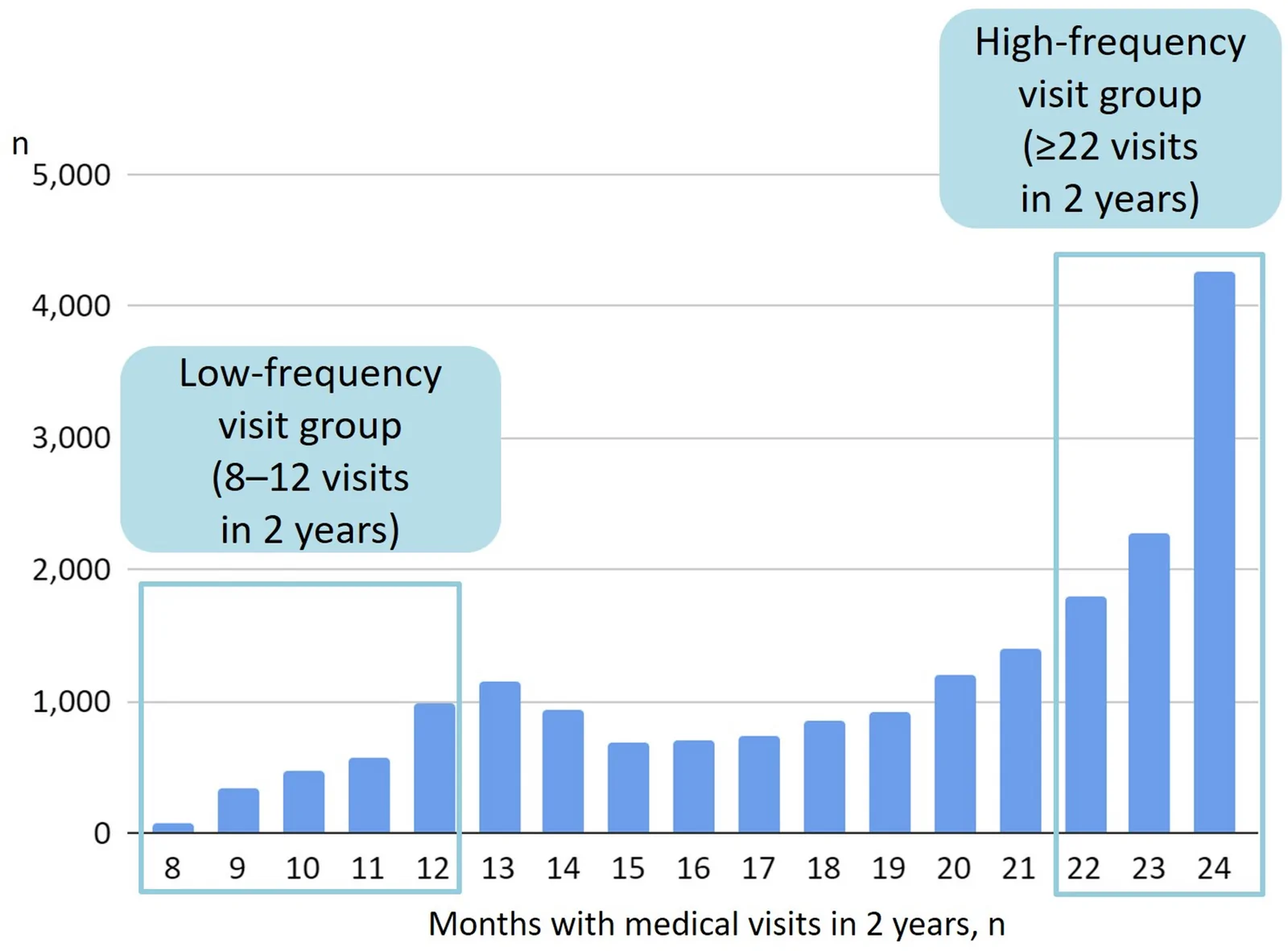
Distribution of the number of months with medical visits during the 2-year exposure period among patients with newly treated dyslipidaemia in the Shizuoka Kokuho Database, Japan (2012–2021)

Figure 2 shows the flow chart for study population selection. A total of 19,364 patients newly initiated dyslipidaemia medication during the study period; of these, 8,344 made ≥ 22 visits and 2,435 made 8–12 visits over 2 years (Fig. 3). Baseline characteristics before and after propensity score matching are shown in Supplementary Table S5 and Table S6. Before matching, patients in the high-frequency visit group were more likely to be female, older, and hypertensive. Health checkup data also showed that they were less likely to consume alcohol and had lower haemoglobin and LDL-C levels. Table 1 shows the baseline characteristics of the matched study population after exclusion of patients who experienced events within 2 years after the index date. After propensity score matching, all variables were well balanced between groups (standardized mean difference < 0.1 in Table 1 and Table S6). Pre-treatment LDL-C levels were 159.98 ± 30.00 mg/dL in the high-frequency visit group and 159.18 ± 29.87 mg/dL in the low-frequency visit group. LDL-C levels within 1 year after treatment initiation were available from health checkup data for 3,283 individuals (74.4%) in the high-frequency group and 1,028 (71.6%) in the low-frequency group. LDL-C levels were 114.39 ± 29.94 mg/dL and 113.64 ± 29.02 mg/dL, respectively, with no significant between-group difference (*p* = 0.478).

**Table 1:** Baseline characteristics of patients with newly treated dyslipidaemia in the Shizuoka Kokuho Database, Japan (2012–2021), according to frequency of medical visits after propensity score matching and exclusion of events within 2 years

Variable	After PSM and exclusion of those with events within 2 years		SMD
	Frequency of medical visits in 2 years		
	≥ 22 visits	8–12 visits	
	(*n* = 4,412)	(*n* = 1,435)	
Sex (female)	3,091 (70.1)	959 (66.8)	0.070
Mean age (years)	67.5 ± 6.95	67.47 ± 6.88	0.013
Age group (years)			
40–49	79 (1.8)	24 (1.7)	0.023
50–59	351 (8.0)	116 (8.1)	
60–69	2,350 (53.3)	775 (54.0)	
70–79	1,452 (32.9)	466 (32.5)	
≥80	180 (4.1)	54 (3.8)	
**Comorbidities**			
Hypertension	1,952 (44.2)	632 (44.0)	0.004
Diabetes mellitus	145 (3.3)	50 (3.5)	0.011
Cardiac arrhythmias	287 (6.5)	94 (6.6)	0.002
Valvular disease	70 (1.6)	23 (1.6)	0.001
Renal disease	79 (1.8)	33 (2.3)	0.036
Liver disease	487 (11.0)	169 (11.8)	0.023
Peptic ulcer	470 (10.7)	161 (11.2)	0.018
Rheumatic disease	112 (2.5)	40 (2.8)	0.015
Hypothyroidism	78 (1.8)	30 (2.1)	0.023
Dementia	24 (0.5)	9 (0.6)	0.011
Depression	177 (4.0)	61 (4.3)	0.012
Psychosis	46 (1.0)	16 (1.1)	0.007
Other neurological disorder	72 (1.6)	28 (2.0)	0.024
Iron/B12 deficiency anemia	107 (2.4)	36 (2.5)	0.005
**Medical checkup data**			
Current smoker	384 (8.7)	137 (9.5)	0.029
Alcohol consumption			
Daily	694 (15.7)	235 (16.4)	0.021
Sometimes	901 (20.4)	297 (20.7)	
Rarely	2,817 (63.8)	903 (62.9)	
Body mass index*	22.82 ± 3.20	22.92 ± 3.29	0.029
Systolic BP (mmHg)	131.07 ± 17.05	130.95 ± 17.12	0.007
AST (U/L)	23.49 ± 7.88	23.36 ± 8.02	0.017
ALT (U/L)	20.71 ± 13.12	20.83 ± 13.32	0.009
γ-GTP (U/L)	31.87 ± 35.46	32.37 ± 34.68	0.014
Uric acid (mg/dL)	5.24 ± 1.30	5.27 ± 1.29	0.025
Creatinine (mg/dL)	0.74 ± 0.20	0.74 ± 0.19	0.046
Haemoglobin (g/dL)	13.79 ± 1.30	13.88 ± 1.29	0.074
HbA_1c_ (%)	5.74 ± 0.66	5.76 ± 0.76	0.031
HDL-C (mg/dL)	62.25 ± 16.00	61.41 ± 16.15	0.052
LDL-C (mg/dL)	159.98 ± 30.00	159.18 ± 29.87	0.027
Triglycerides (mg/dL)	135.42 ± 93.39	134.36 ± 84.26	0.012
ASCVD risk**			
Low	333 (7.5)	100 (7.0)	0.047
Medium	3,222 (73.0)	1,039 (72.4)	
High	677 (15.3)	242 (16.9)	
≥ 80 years	180 (4.1)	54 (3.8)	
**Medication**			
Statin	3,916 (88.8)	1,273 (88.7)	0.001
Fibrate	250 (5.7)	80 (5.6)	0.004
Ezetimibe	124 (2.8)	39 (2.7)	0.006

The data are presented as the number (percentage) or as the mean ± standard deviation as appropriate. *Calculated as kg/m^2^. **Risk based on the Hisayama score in patients younger than 80 years. ALT, alanine aminotransferase; ASCVD, atherosclerotic cardiovascular disease; AST, aspartate aminotransferase; BP, blood pressure; GTP, glutamyl transpeptidase; HDL-C, high-density lipoprotein cholesterol; LDL-C, low-density lipoprotein cholesterol; PSM, propensity score matching; SMD, standardized mean difference

### Comparison of cardiac events, cerebrovascular events, and mortality

**Fig. 4:**
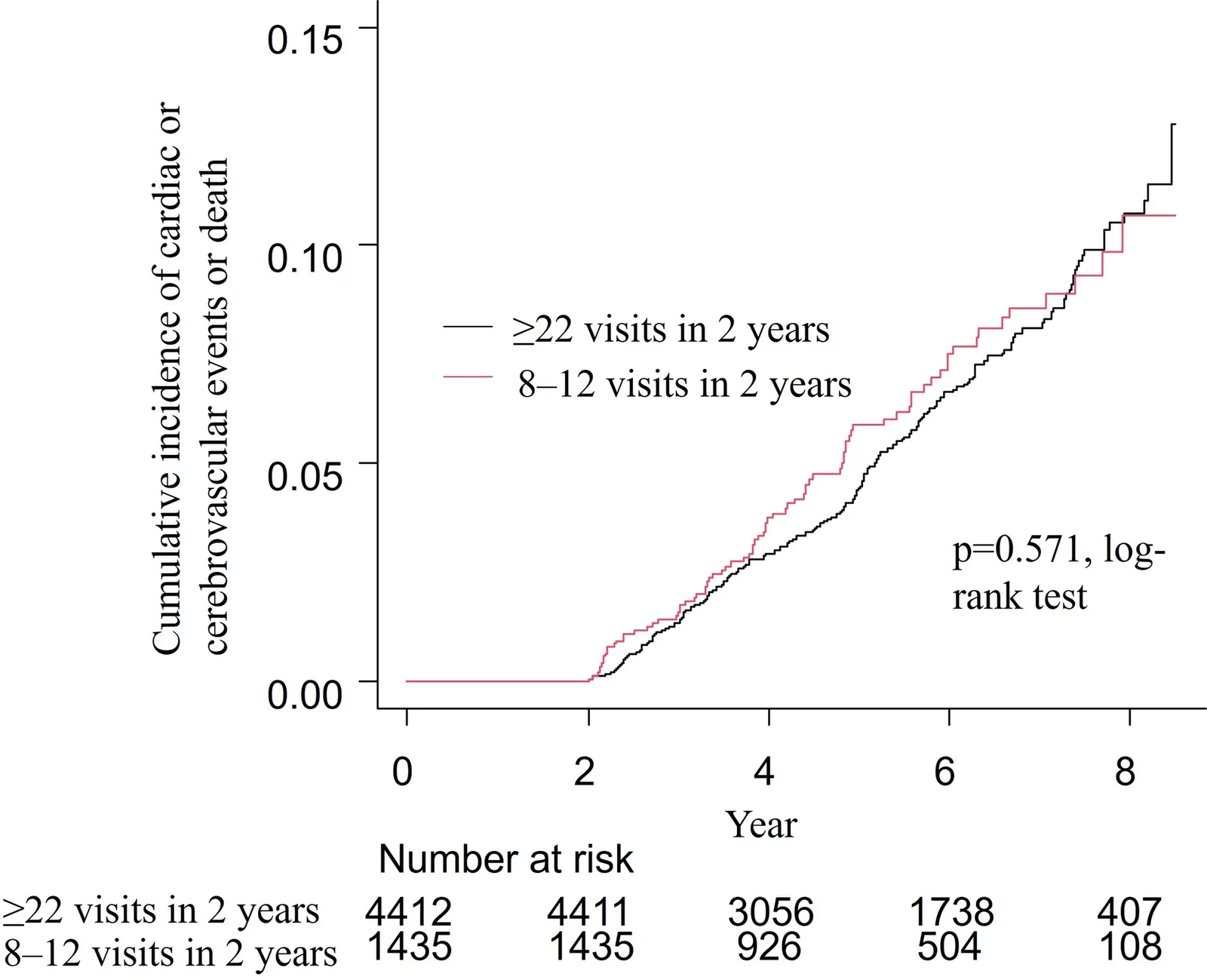
Cumulative incidence of cardiac events, cerebrovascular events, or death according to visit frequency among patients with newly treated dyslipidaemia in the Shizuoka Kokuho Database, Japan (2012–2021)n

The 1:3 matched cohort included 1,435 patients in the low-frequency visit group and 4,412 in the high-frequency visit group. Composite outcomes occurred in 81 patients (5.6%) and 249 patients (5.6%), respectively (Table 2). The hazard ratio (HR) for the low-frequency visit group relative to the high-frequency visit group was 1.08 (95% CI 0.84–1.38; *p* = 0.571), indicating no statistically significant difference (Table 2; Fig. 4).

**Table 2:** Comparison of cardiovascular outcomes and mortality by visit frequency among patients with newly treated dyslipidaemia in the Shizuoka Kokuho Database, Japan (2012–2021), after propensity score matching

Outcome	Medical visits in 2 years (n)	Events, n (%)	Cumulative incidence at 5 years		p-value
			Rate	95% CI	
Composite of events^**†**^	8–12	81 (5.6)	5.9	4.6–7.5	0.571
	≥ 22	249 (5.6)	4.4	3.8–5.2	
Cardiac events*	8–12	54 (3.8)	4.0	2.9–5.3	0.440
	≥ 22	158 (3.6)	2.8	2.3–3.4	
Cerebrovascular events*	8–12	19 (1.3)	1.2	0.7–2.0	0.647
	≥ 22	55 (1.2)	1.0	0.7–1.4	
Death^**†**^	8–12	24 (1.7)	1.3	0.8–2.3	0.790
	≥ 22	84 (1.9)	1.4	1.1–1.9	

There were 1,435 patients in the low-frequency visit group and 4,412 in the high-frequency visit group. *Gray’s test. ^†^Log-rank test. CI, confidence interval

For individual outcomes, cardiac events occurred in 54 patients (3.8%) in the low-frequency visit group and 158 (3.6%) in the high-frequency visit group (HR 1.13; 95% CI 0.83–1.54), while cerebrovascular events occurred in 19 (1.3%) and 55 (1.2%), respectively (HR 1.13; 95% CI 0.67–1.90). Death occurred in 24 patients (1.7%) in the low-frequency visit group and 84 (1.9%) in the high-frequency visit group (HR 0.94; 95% CI 0.60–1.48). No statistically significant between-group difference was observed for any individual outcome (Table 2 and Supplementary Figure S1).

### Sensitivity analysis

First, groups were compared according to the number of visits during the second year of the 2-year period, with the low-frequency visit group defined as 4–6 visits and the high-frequency visit group as ≥ 11 visits (Supplementary Figure S2). The p-value for the composite outcome was 0.690, indicating no statistically significant difference between the groups (Supplementary Table S7 and Figure S3).

Next, groups were compared according to the cumulative number of visits over the 2-year period, with the low-frequency visit group expanded from 8 to 12 to 8–16 visits (Supplementary Figure S4). In this analysis, the p-value for the composite outcome was 0.854, again indicating no significant difference between the groups (Supplementary Table S8 and Figure S5).

Finally, patients with 13–21 visits over the 2-year period were categorized as an intermediate-frequency group and compared with both the low-frequency and high-frequency visit groups (Supplementary Figure S6). The p-value for the composite outcome was 0.669 for the comparison between the low-frequency and intermediate-frequency groups and 0.897 for that between the intermediate-frequency and high-frequency groups, indicating no significant difference in either comparison (Supplementary Table S9 and Figure S7; Supplementary Table S10 and Figure S8).

### Subgroup analysis

Supplementary Figure S9 presents the results of the subgroup analysis, including the HRs and interaction p-values for cardiac events, cerebrovascular events, and mortality. No statistically significant interaction was observed in any subgroup, indicating no evidence of effect heterogeneity.

## Discussion

This retrospective cohort study used data from the SKDB in Japan to examine the association between the frequency of medical visits after initiation of lipid-lowering therapy and the long-term risks of cardiac events, cerebrovascular events, and mortality. We found no significant difference in the primary outcome across intervals between visits. To our knowledge, this is the first study to investigate the association between visit interval and long-term outcomes in patients with dyslipidaemia. In Japan, more frequent medical visits have been reported to be associated with better blood pressure control in patients with hypertension [21], and a similar finding has been reported in the USA [22]. By contrast, a nationwide study from South Korea, another country with one of the highest annual medical visit rates in the world, reported better long-term outcomes in patients with hypertension when the interval between visits was longer [23]. Thus, findings on the association between visit interval and health outcomes in chronic disease remain inconsistent, and the optimal frequency of medical visits has not been established. In this context, our study provides novel insights not only for countries such as Japan and South Korea, where visit frequency is high, but also for the broader global context. Because dyslipidaemia is commonly managed in primary care, these findings may be relevant to follow-up strategies in routine primary care practice beyond Japan. While many claims-based studies suffer from the lack of lifestyle information and clinical laboratory data, our study was able to adjust for important confounders, including smoking status, alcohol consumption, body mass index, and laboratory test results, through integration with health checkup data. This represents a major methodological strength of the present study and may have reduced residual confounding compared with studies based solely on administrative claims databases.

The high frequency of medical visits in Japan may be attributable to its universal health insurance system, which features low copayments and free access to healthcare, thereby lowering barriers to medical consultation [24]. Japan is also one of the world’s leading countries in longevity, and its high accessibility to healthcare may contribute to increasing life expectancy [25]. Nevertheless, our study did not show that more frequent visits were associated with fewer cardiac events or deaths among patients newly prescribed lipid-lowering agents. A previous study examining the effectiveness of statins in specific subgroups reported greater benefit among older adults and patients with diabetes [26]. However, we found no significant difference in the effect of visit frequency across subgroups. These findings suggest that although medication itself may help prevent events, more frequent visits do not necessarily confer additional risk reduction in patients with dyslipidaemia who have no history of ASCVD. Importantly, treatment was still continued even in the low-frequency visit group. Current ESC/EAS and JAS guidelines recommend that the intensity of lipid-lowering management be determined according to baseline cardiovascular risk and treatment response. Therefore, decisions regarding the extension of visit intervals should be individualized, particularly for patients at high cardiovascular risk or those requiring treatment intensification [3, 17].

One possible explanation for a higher frequency of medical visits is inadequate improvement in LDL-C levels or the need for more intensive lipid-lowering management. However, as noted in the Results, LDL-C levels within 1 year after treatment initiation did not differ significantly between the two groups, and the use of statins and ezetimibe was well balanced after propensity score matching. Nevertheless, from the perspective of treat-to-target lipid management, comparable achieved LDL-C levels do not necessarily indicate comparable treatment intensity or underlying disease severity. Differences in medication dosage, dose escalation, the timing of add-on therapy, or the need for closer monitoring may have contributed to the observed visit-frequency patterns. Because detailed information on medication dosage, dose adjustment, and the timing of treatment intensification was unavailable, these possibilities could not be fully evaluated. In addition, there was a slight between-group difference in the proportion of individuals with available health checkup data after treatment initiation. Nevertheless, as shown in the sensitivity analysis, no significant between-group differences in outcomes were observed even when patients were grouped according to the number of visits in the second year after 1 year of treatment. Because LDL-C values were obtained from annual health checkup data, we were able to evaluate LDL-C levels only at baseline and at approximately 1 year after treatment initiation. Therefore, we could not assess detailed longitudinal changes in LDL-C levels throughout the 2-year exposure assessment period. Future studies linking claims data with laboratory results obtained during routine clinical practice may allow a more detailed evaluation of LDL-C trajectories, treatment intensification, and treatment response over time.

As noted in the Introduction, a refill prescription system was introduced in Japan in 2022 for patients with stable chronic disease, allowing physician-issued prescriptions to be used up to three times [11]. For example, a 30-day prescription can be reused twice, providing a total of 90 days of medication with a single visit. Likewise, a 90-day prescription can cover up to 270 days with one visit. However, the uptake of refill prescriptions remains below 1% more than 2 years after implementation [27, 28]. Wider use of refill prescriptions is expected to reduce healthcare costs and clinical workload, thereby allowing clinicians to devote more time to complex diagnoses, specialized treatments, management of severe disease, in-depth consultations, and staying abreast of medical advances [29, 30]. In our study, the long-term risks of cardiac and cerebrovascular events did not differ significantly between patients visiting monthly and those visiting every 2–3 months after initiation of lipid-lowering therapy, suggesting that extending visit intervals may be a reasonable option for some selected stable patients. This finding may provide greater reassurance for both patients and physicians in using refill prescriptions. Although no statistically significant difference was observed, the 95% confidence interval for the composite outcome was relatively wide and included a hazard ratio as high as 1.38. Therefore, a clinically meaningful increase in risk associated with less frequent visits cannot be completely excluded, and the findings should not be interpreted as demonstrating equivalence or non-inferiority between the visit-frequency groups.

### Limitations

This study has several limitations. First, group allocation was based on prescription duration, and actual medication adherence was unknown. Some patients visiting every 2–3 months may have had extended visit intervals because of poor adherence despite receiving a 1-month prescription. Accounting more rigorously for adherence might therefore have yielded more robust results. Second, visit frequency was assessed only during the first 2 years after the initial prescription, and subsequent visits were not tracked. Some crossover from the high-frequency to the low-frequency visit group would be expected over time. If visit frequency or interval affects outcomes, incorporating visits beyond 2 years might attenuate between-group differences. Third, although this study was informed by the concept of a new-user design, we did not explicitly emulate a target trial or apply a clone-censor-weight (CCW) approach to align eligibility, treatment assignment, and follow-up under alternative visit-frequency strategies. Thus, time-related biases, including immortal time bias and informative censoring, cannot be fully excluded, and causal interpretation should remain cautious. Fourth, unmeasured contextual factors, including place of residence, patient–physician relationship, patient preferences, healthcare facility characteristics, and physician characteristics, may have influenced follow-up patterns. The influence of facility type could not be directly assessed because facility-level analyses were restricted by the database-use conditions. Therefore, differences in follow-up practices across healthcare facilities, as well as clinical considerations regarding disease stability and perceived patient risk, may have contributed to residual confounding. Fifth, because the population covered by the SKDB may differ from populations covered by employment-based health insurance, the generalizability of these findings to other insured populations should be interpreted with caution. Sixth, the individual cardiac outcome was limited to acute coronary syndrome and heart failure. Sudden cardiac death could not be identified as a separate cause-specific outcome because cause-of-death information was unavailable. Severe arrhythmias were not assessed separately because their clinical severity and acuity could not be reliably determined using claims data alone. Finally, this study was limited to patients with dyslipidaemia without a history of cardiac or cerebrovascular events, and the event incidence was low. The annual incidence of cardiac events among statin users in the primary prevention setting has been reported to be 1.3%, similar to that observed in our study [31]. Since patients undergoing secondary prevention typically require more intensive monitoring and lipid management, these findings cannot be generalized to secondary-prevention populations. Future studies with longer follow-up and broader populations, including patients with other chronic diseases or those undergoing secondary prevention, are warranted.

## Conclusion

This study found no significant association between the frequency of medical visits and the incidence of cardiac events, cerebrovascular events, or mortality in patients with dyslipidaemia. These findings suggest that, for selected stable patients receiving lipid-lowering therapy for the primary prevention of cardiovascular and cerebrovascular events, extending visit intervals to 2–3 months may be a reasonable option. Future studies should extend the follow-up period and include patients with other chronic diseases and those undergoing secondary prevention to confirm these findings and inform evidence-based policies on visit intervals.

## Supplementary Information

Supplementary material 1.

## Data Availability

According to the terms of Shizuoka Prefecture’s data use agreement with local insurers, the analysed data cannot be provided to readers by the authors. Researchers interested in accessing this dataset may apply to Shizuoka Prefecture to request access. Please contact the staff of Shizuoka Graduate University of Public Health (e-mail: info@s-sph.ac.jp).
