# Supplementary material for: Association of medical visit frequency with cardiovascular events and mortality in patients with dyslipidaemia: a cohort study using a Japanese claims database
Source: J Health Popul Nutr. 2026 Aug 2;45:241. doi: 10.1186/s41043-026-01408-5 (PMC13721620; doi:10.1186/s41043-026-01408-5)
Supplement: Supplementary file 1 [file 41043_2026_1408_MOESM1_ESM.docx]

**Supporting information**

**Supplementary Figure S1. Cumulative incidence of cardiac events, cerebrovascular events, and mortality by frequency of visits in 2 years.**

**a. Cardiac events	b. Cerebrovascular events**

**c. Mortality**

This figure compares the cumulative incidence of (a) cardiac events, (b) cerebrovascular events, and (c) mortality between patients who made ≥22 medical visits in 2 years (the high-frequency visit group) and those who made 8–12 visits in 2 years (the low-frequency visit group). There was no statistically significant between-group difference in the cumulative incidence of cardiac events (p=0.440), cerebrovascular events (p=0.647), or mortality (p=0.790). The numbers of subjects at risk are also shown at various time points.

**Supplementary Figure S2. Distribution of medical visits in the second year of 2 years.**

This figure compares the distribution of medical visits in the second year between the low-frequency visit group (4–6 visits) and the high-frequency visit group (≥11 visits). The x-axis represents the number of medical visits per year, while the y-axis shows the number of subjects in each group.

**Supplementary Figure S3. Cumulative incidence of the composite outcome by frequency of visits in the second year of 2 years.**

This figure compares the cumulative incidence of the composite outcome between the high-frequency visit group (≥11 visits in the second year) and the low-frequency visit group (4–6 visits in the second year). The cumulative incidence of the composite outcome (p=0.690) indicates that there were no statistically significant between-group differences. The number of subjects at risk at various time points are also shown.

**Supplementary Figure S4. Distribution of medical visits over a 2-year period. (redefinition of the low-frequency visit group).**

This figure compares the distribution of medical visits between the high-frequency visit group (≥22 visits over 2 years) and the redefined low-frequency visit group (when defined as 8–16 visits over 2 years). The x-axis represents the number of medical visits over the 2-year period and the y-axis shows the number of subjects in each group.

**Supplementary Figure S5. Cumulative incidence of the composite outcome with redefinition of the low-frequency visit group (8–12 to 8–16 visits over 2 years)**

This figure compares the cumulative incidence of the composite outcome between patients with a high frequency of visits (≥22 over 2 years) and those with a redefined low frequency of visits (8–16 over 2 years). There was no statistically significant between-group difference in the cumulative incidence of the composite outcome (p=0.854). The numbers of patients at risk are also shown at various time points.

**Supplementary Figure S6. Distribution of medical visits over a 2-year period (addition of the intermediate-frequency visit group).**

This figure compares the distribution of medical visits among the intermediate-frequency visit group (13–21 visits over 2 years), the high-frequency visit group (≥22 visits over 2 years), and the low-frequency visit group (8–12 visits over 2 years). The x-axis represents the number of medical visits over the 2-year period, while the y-axis shows the number of subjects in each group.

**Supplementary Figure S7. Comparison of the cumulative incidence of the composite outcome between the low-frequency and intermediate-frequency visit groups**

This figure compares the cumulative incidence of the composite outcome between patients with a low visit frequency (8–12 visits over 2 years) and those with an intermediate visit frequency (13–21 visits over 2 years). There was no statistically significant between-group difference in the cumulative incidence of the composite outcome (p=0.669). The number of patients at risk is also shown at various time points.

**Supplementary Figure S8. Comparison of the cumulative incidence of the composite outcome between the intermediate-frequency and high-frequency visit groups**

This figure compares the cumulative incidence of the composite outcome between patients with an intermediate visit frequency (13–21 visits over 2 years) and those with a high visit frequency (≥22 visits over 2 years). There was no statistically significant between-group difference in the cumulative incidence of the composite outcome (p=0.897). The number of patients at risk is also shown at various time points.

**Supplementary Figure S9.** **Subgroup analysis of the composite outcome.**

This figure presents the HRs and 95% CIs for the composite outcome between patients with a high frequency of visits (≥22 visits over 2 years) and those with a low frequency of visits (8–12 visits over 2 years). ASCVD, atherosclerotic cardiovascular disease; CI, confidence interval; HR, hazard ratio.

**Supplementary Table S1.** Dyslipidemia-related diagnoses and corresponding ICD-10 codes in Japan

| **Disease code** | **ICD-10** | **Disease name** |
| --- | --- | --- |
| 2720004 | E780 | Hypercholesterolemia |
| 2724036 | E780 | High LDL-C |
| 8840108 | E780 | Essential hypercholesterolemia |
| 8851070 | E780 | High LDL-C |
| 2721002 | E781 | Hypertriglyceridemia |
| 8833881 | E782 | Mixed hyperlipidemia |
| 2724023 | E784 | Dietary hyperlipidemia |
| 2724007 | E785 | Hyperlipidemia |
| 2724012 | E785 | Essential hyperlipidemia |
| 8844446 | E785 | Dyslipidemia |
| 2729002 | E789 | Disorders of lipoprotein metabolism |

HDL-C, high-density lipoprotein cholesterol; ICD-10, International Classification of Diseases, 10th Revision

**Supplementary Table S2.** Drug codes for various agents used in the treatment of dyslipidemia categorized by type, including statins, fibrates, ezetimibe, and others

|  | **Codes** |
| --- | --- |
| Statins | 610443013, 610443014, 622076401, 622076501, 622107601, 622475000, 622107701, 622126901, 622127001, 622475100, 622098401, 622098501, 622110401, 622110501, 622161801, 622161901, 622143801, 622143901, 622152001, 622152101, 622170101, 622170201, 622165601, 622475000, 622165701, 622475100, 622217101, 622475000, 622217201, 622187601, 622187701, 622204801, 622204901, 622186601, 622475000, 622186701, 622475100, 622241301, 622241401, 622244801, 622244901, 622244801, 622244901, 622252001, 622252101, 622252001, 622252101, 622302401, 622475000, 622302501, 622475100, 622280701, 622280801, 622167601, 622475000, 622167701, 622475100, 622360101, 622180602, 622180702, 622180602, 622180702, 622180602, 622180702, 622180602, 622180702, 612180263, 610462015, 610462016, 620000051, 620000169, 620000054, 620000175, 620002166, 620000054, 620002049, 620002050, 620002162, 620000177, 620002164, 620000051, 620000174, 620002880, 620000177, 620002164, 620000054, 620002049, 620002050, 620009322, 620009323, 622315500, 620009325, 620000053, 620002736, 622315500, 620009324, 620000053, 620002736, 620009324, 620000052, 622315400, 621948701, 622315500, 621948801, 620000178, 621955001, 621955101, 620000178, 621955001, 620000178, 620000179, 620002165, 622055601, 622071601, 622128201, 622128301, 621643301, 622099101, 622099201, 622075801, 622075901, 621534101, 621643401, 622052801, 620000176, 620002163, 622102501, 620000051, 622116801, 622116901, 621534101, 621643401, 620000178, 621955001, 621534301, 621643501, 622055602, 621533901, 622315400, 622128201, 622315500, 622128301, 621533801, 621675101, 622071601, 621524102, 622116802, 622315500, 622116902, 622691000, 621524102, 621524102, 621524402, 622315400, 621635202, 622315500, 621635301, 621524402, 622315400, 621635202, 622315500, 621635301, 621643601, 620000176, 622315400, 620002163, 622102501, 621534204, 622315400, 621955003, 622315500, 621534204, 622315400, 621955003, 622315500, 621534204, 622315400, 621533501, 622169902, 622170002, 621534003, 622315400, 621623603, 622102502, 622136401, 620000422, 620000423, 622615700, 622615800, 622427801, 622419701, 622419801, 622419901, 620000422, 620000423, 622136401, 622273101, 622615600, 622273201, 622691800, 622273301, 622291801, 622291901, 622292001, 622276301, 622615600, 622276401, 622691800, 622276501, 622615900, 622239201, 622239301, 622287601, 622292301, 622615600, 622292401, 622691800, 622292501, 622296001, 622296101, 622296201, 622280201, 622280301, 622280401, 622294301, 622615600, 622294401, 622691800, 622294501, 622615900, 622286201, 622286301, 622286401, 622268001, 622268101, 622268201, 622615900, 622274901, 622615600, 622275001, 622691800, 622275101, 622298001, 622298101, 622298201, 622299001, 622299101, 622359101, 622304601, 622304701, 622362701, 622615900, 622280501, 622280601, 622372401, 622283701, 622283801, 622342801, 622293301, 622293401, 622392501, 622269101, 622615600, 622269201, 622691800, 622365801, 622271801, 622615600, 622271901, 622691800, 622387601, 622434301, 622434401, 622297101, 622297201, 622427801, 622419701, 622419801, 622419901, 622421601, 622421701, 622421801, 622285001, 622285101, 622347401, 622304801, 622304901, 622427701, 622615900, 622302801, 622302901, 622387001, 622615900, 622282201, 622282301, 622691800, 622426201, 622289501, 622289601, 622431401, 622457701, 622457801, 622457901, 622270001, 622615600, 622270101, 622691800, 622406901, 622615900, 622464901, 622465001, 622691800, 622465101, 622615900, 622522101, 622615700, 622522201, 622615800, 622522301, 622524501, 622524601, 622524701, 622512001, 622615700, 622512101, 622615800, 622512201, 622270001, 622615600, 622270101, 622691800, 622406901, 622615900, 622528901, 622529001, 622568601, 622604001, 622604101, 622604201, 622665901, 622666001, 622666101, 622297101, 622297201, 610454084, 612180264, 610454085, 612180265, 620000038, 620000039, 620000111, 620000112, 620000140, 620000141, 620000156, 620000157, 620000103, 620000104, 620000109, 620000110, 620000156, 620000157, 620000163, 620000164, 620000013, 620000014, 620000105, 620000106, 620000159, 620000160, 620000113, 620000114, 620000070, 620000071, 620000109, 620000110, 620000107, 620000108, 620002108, 620002109, 620002117, 620002118, 620002879, 620000171, 620002801, 620002802, 620002798, 620002799, 620002798, 620002799, 620000151, 620000152, 620000153, 620002800, 622321900, 620004038, 620004085, 620000158, 620008055, 622321900, 620008056, 622139600, 620008053, 622321900, 620008054, 622139600, 620000107, 620000108, 620000070, 620000071, 621981401, 620006681, 622062701, 620000151, 620000152, 620000153, 621639701, 621639801, 621528801, 621528901, 620002801, 620002802, 621531001, 621531101, 621528602, 621528702, 621528602, 621528702, 621533101, 621533201, 621529001, 622321900, 621529101, 621639001, 622321900, 621639101, 622139600, 621525701, 621525801, 621694001, 621533601, 621523101, 621523201, 621532902, 622321900, 621533002, 622139600, 621532401, 621532501, 621532401, 621532501, 621981403, 621531703, 621521301, 621521401, 622321900, 622606601, 622139600, 622606701, 621752501, 621532601, 622139600, 620000105, 620000106, 621528401, 621528501, 610470012, 610470013, 610470014, 621964101, 621964301, 621964501, 621934801, 621934901, 621935001, 621964201, 621964401, 621964601, 622015101, 622015201, 622015301, 620002477, 620002478, 620002477, 620002478, 620002477, 620002478, 622441101, 622441201, 620002477, 620002478, 622441101, 622441201, 622600301, 622692400, 622605301, 622692600, 622605001, 622692500, 622605101, 622692700, 622592501, 622692400, 622592601, 622692600, 622592701, 622692500, 622592801, 622692700, 622578401, 622578501, 622578701, 622578801, 622578601, 622598301, 622692400, 622598401, 622692600, 622598501, 622692500, 622598601, 622692700, 622537301, 622692400, 622537401, 622692600, 622591701, 622591801, 622591901, 622592001, 622582001, 622582101, 622581901, 622581701, 622581801, 622581601, 622592901, 622692400, 622593001, 622692600, 622593101, 622692500, 622593201, 622692700, 622600601, 622692400, 622600701, 622692600, 622600801, 622692500, 622600901, 622692700, 622590301, 622692400, 622590401, 622692600, 622577901, 622578001, 622577901, 622578001, 622599201, 622692400, 622599301, 622692600, 622578101, 622578201, 622578101, 622578201, 622572801, 622692400, 622572901, 622692600, 622590101, 622692400, 622590201, 622692600, 622601201, 622601301, 622601401, 622601501, 622586001, 622586101, 622586201, 622586301, 622586201, 622586301, 622575201, 622692400, 622575301, 622692600, 622575401, 622692500, 622575501, 622599401, 622692500, 622599501, 622692700, 622605201, 622605401, 622584701, 622692400, 622584801, 622692600, 622588801, 622692400, 622588901, 622692600, 622589001, 622692500, 622589101, 622692700, 622595301, 622692400, 622595401, 622692600, 622582501, 622582701, 622582601, 622582801, 622644801, 622644901, 622640801, 622692400, 622640901, 622692600, 622571801, 622571901, 622575601, 622575701, 622660001, 622692400, 622660101, 622692600, 622605201, 622692400, 622605401, 622692600, 622644801, 622692500, 622644901, 622692700, 622692400, 622803401, 622692600, 622803501, 622692500, 622803601, 622692700, 622803701, 622692500, 622803601, 622692700, 622803701 |
| Fibrates | 610422276, 610422259, 610422258, 610422265, 620001883, 622728600, 610422264, 622322900, 622728700, 610422263, 610422262, 610433126, 620001883, 610433125, 610422259, 610422276, 620002123, 610407028, 620002433, 620002434, 620002433, 620002434, 620002423, 620002424, 620002541, 620002540, 620008508, 622322900, 622728700, 612180028, 620009218, 612180028, 620338317, 620001883, 620339401, 622728600, 612180106, 610433008, 620001883, 622026701, 612180028, 620338305, 622039501, 622039601, 620001883, 620340602, 622096101, 622096801, 622096901, 622090701, 622090801, 622090701, 622090801, 620001883, 620339201, 622728600, 620340901, 622322900, 622728700, 620001883, 620339201, 622728600, 610422276, 620001883, 622728600, 622223601, 622322900, 622728700, 620001883, 620340603, 622728600, 622096102, 622322900, 622728700, 620001883, 620340201, 622728600, 620341301, 622322900, 622728700, 620001883, 621254601, 622728600, 622026702, 622322900, 622728700, 622573101, 622590501, 622590601, 620339501, 622728600, 620341001, 622728700, 620339603, 622728600, 620341102, 622728700 |
| Ezetimibe | 620004868, 622704001, 622801101, 622808201, 622846700, 622797701, 622814501, 622805001, 622809701, 622786001, 622790901, 622794301, 622799601, 622789601, 622789701, 622806401, 622704101, 622791901, 622795201, 622812801, 622784901, 622792901 |
| Others | 622739100, 622739000, 622738900, 622728900, 622728800, 622610500, 622579101, 622516901, 622516801, 622516701, 622508501, 622508401, 622488901, 622471801, 622468701, 622468601, 622468501, 621960102, 621960101, 621960002, 621960001, 621959902, 621959901, 621869705, 621869605, 621869505, 621776502, 621315603, 620817702, 620817504, 620817103, 620817001, 620816805, 620816501, 620816301, 620816201, 620815903, 620815601, 620815302, 620691102, 620691002, 620690701, 620689802, 620673518, 620673513, 620673501, 620672920, 620672914, 620672913, 620346101, 620346029, 620346023, 620346018, 620346008, 620346001, 620344306, 620009208, 620008784, 620008767, 620008766, 620008761, 620008706, 620008631, 620008610, 620008294, 620008113, 620008112, 620008111, 620007995, 620007994, 620007993, 620007898, 620007897, 620007896, 620007895, 620007894, 620007893, 620007892, 620007891, 620007890, 620007889, 620007888, 620007887, 620006870, 620006574, 620006115, 620006108, 620005965, 620005932, 620005920, 620005785, 620005513, 620005032, 620004547, 620004546, 620004519, 620004459, 620003669, 620003668, 620003623, 620003597, 620003596, 620003595, 620002508, 620001924, 613390006, 613130166, 613130165, 612900013, 612180292, 612180141, 612180140, 612180004, 610463152, 610463151, 610463087, 610462007, 610461121, 610432003, 610422292, 610422290, 610422286, 610422221, 610422206, 610422123, 610422098, 610422059, 610422057, 610422056, 610422055, 610422054, 610422031, 610422023, 610422004, 610421344, 610421343 |

**Supplementary Table S3.** Definitions of various outcomes, including the corresponding ICD-10 or disease codes and details

| **Outcome** | **ICD-10 or disease code** | **Details** |
| --- | --- | --- |
| Cardiac event | I20, I21, and I22 (acute coronary syndromes)  I50 (heart failure) | Only participants with these codes who were hospitalized were considered to have experienced events |
|  | 150260350, 150284310, 150318310, 150359310, 150153910, 150374910, 150375010, 150375110, 150263310, 150375210, 150375310, 150375410, 150145910, 150146010, 150318410, 150318510 | Percutaneous coronary intervention or coronary artery bypass grafting |
| Cerebrovascular event | I60, I61, I63, and I64 (stroke)  8838736, 8838748, and 8838750 (other cerebrovascular event) | Only patients with these codes who were hospitalized were considered to have experienced events |

ICD-10, International Classification of Diseases, 10th Revision

**Supplementary Table S4.** Comorbidities and their corresponding ICD-10 codes

| **Comorbidity** | **ICD-10 codes** |
| --- | --- |
| Hypertension | I10, I11, I12, I13, I15 |
| Diabetes | E100, E101, E102, E103, E104, E105, E106, E107, E108, E109, E110, E111, E112, E113, E114, E115, E116, E117, E118, E119, E120, E121, E122, E123, E124, E125, E126, E127, E128, E129, E130, E131, E132, E133, E134, E135, E136, E137, E138, E139, E140, E141, E142, E143, E144, E145, E146, E147, E148, E149 |
| Cardiac arrhythmias | I441, I442, I443, I456, I459, I47, I48, I49, R000, R001, R008, T821, Z450, Z950 |
| Valvular disease | A520, I05, I06, I07, I08, I091, I098, I34, I35, I36, I37, I38, I39, Q230, Q231, Q232, Q233, Z952, Z953, Z954 |
| Chronic kidney disease | I120, I131, N018, N019, N032, N033, N034, N035, N036, N037, N052, N053, N054, N055, N056, N057, N250, Z490, Z491, Z492, Z940, Z992 |
| Liver disease | B18, I85, I850, I859, I864, I982, K700, K701, K702, K703, K704, K709, K711, K713, K714, K715, K717, K721, K729, K73, K74, K760, K762, K763, K764, K765, K766, K767, K768, K769, Z944 |
| Peptic ulcer | K25, K257, K259, K26, K267, K269, K27, K277, K279, K28, K287, K289 |
| Rheumatic disease/collagen vascular diseases | L940, L941, L943, M05, M06, M08, M120, M123, M30, M310, M311, M312, M313, M315, M32, M33, M34, M351, M353, M360, M35, M45, M461, M468, M469 |
| Hypothyroidism | E00, E01, E02, E03, E890 |
| Dementia | F00, F01, F02, F03, F051, G30, G311 |
| Depression | F204, F313, F314, F315, F32, F33, F341, F412, F432 |
| Psychoses | F20, F22, F23, F24, F25, F28, F29, F302, F312, F315 |
| Other neurological disorders | G10, G11, G12, G13, G20, G21, G22, G254, G255, G312, G318, G319, G32, G35, G36, G37, G40, G41, G931, G934, R470, R56 |
| Iron/B12 deficiency anemia | D508, D509, D51, D52, D53 |

ICD-10, International Classification of Diseases, 10th Revision

**Supplementary Table S5.** Baseline characteristics of the study population according to frequency of medical visits before propensity score matching

|  | Variable | Before PSM | | SMD |
| --- | --- | --- | --- | --- |
|  |  | Frequency of medical visits  in 2 years | |  |
|  |  |  |  |  |
|  |  | ≥22 visits | 8–12 visits |  |
|  |  | (n=8,344) | (n=2,435) |  |
|  | Female sex | 5,778 (69.2) | 1,564 (64.2) | 0.107 |
|  | Mean age (years) | 69.74 ± 7.90 | 67.04 ± 7.62 | 0.347 |
|  | Age group (years) |  |  |  |
|  | 40–49 | 140 (1.7) | 71 (2.9) | 0.353 |
|  | 50–59 | 521 (6.2) | 225 (9.2) |  |
|  | 60–69 | 3,490 (41.8) | 1,285 (52.8) |  |
|  | 70–79 | 3,267 (39.2) | 745 (30.6) |  |
|  | ≥80 | 926 (11.1) | 109 (4.5) |  |
|  | **Comorbidities** |  |  |  |
|  | Hypertension | 4,363 (52.3) | 981 (40.3) | 0.242 |
|  | Diabetes mellitus | 302 (3.6) | 92 (3.8) | 0.008 |
|  | Cardiac arrhythmias | 596 (7.1) | 153 (6.3) | 0.034 |
|  | Valvular disease | 175 (2.1) | 34 (1.4) | 0.054 |
|  | Renal disease | 143 (1.7) | 54 (2.2) | 0.036 |
|  | Liver disease | 954 (11.4) | 289 (11.9) | 0.014 |
|  | Peptic ulcer | 861 (10.3) | 275 (11.3) | 0.031 |
|  | Rheumatic disease | 194 (2.3) | 70 (2.9) | 0.035 |
|  | Hypothyroidism | 124 (1.5) | 56 (2.3) | 0.06 |
|  | Dementia | 132 (1.6) | 19 (0.8) | 0.074 |
|  | Depression | 399 (4.8) | 123 (5.1) | 0.012 |
|  | Psychosis | 129 (1.5) | 23 (0.9) | 0.054 |
|  | Other neurological disorder | 167 (2.0) | 42 (1.7) | 0.020 |
|  | Iron/B12 deficiency anemia | 237 (2.8) | 64 (2.6) | 0.013 |
|  | **Medical checkup data** |  |  |  |
|  | Current smoker | 685 (8.2) | 252 (10.3) | 0.074 |
|  | Alcohol consumption |  |  |  |
|  | Daily | 1,079 (15.2) | 374 (17.6) | 0.117 |
|  | Sometimes | 1,304 (18.4) | 456 (21.5) |  |
|  | Rarely | 4,719 (66.4) | 1,290 (60.8) |  |
|  | Body mass index* | 22.97 ± 3.26 | 22.88 ± 3.32 | 0.027 |
|  | Systolic BP (mmHg) | 133.50 ± 17.45 | 129.56 ± 17.31 | 0.227 |
|  | AST (U/L) | 23.91 ± 9.33 | 23.33 ± 7.81 | 0.068 |
|  | ALT (U/L) | 20.52 ± 12.92 | 21.09 ± 13.63 | 0.043 |
|  | γ-GTP (U/L) | 31.52 ± 35.65 | 32.63 ± 35.17 | 0.031 |
|  | Uric acid (mg/dL) | 5.25 ± 1.30 | 5.35 ± 1.33 | 0.074 |
|  | Creatinine (mg/dL) | 0.74 ± 0.21 | 0.75 ± 0.19 | 0.053 |
|  | Hemoglobin (g/dL) | 13.70 ± 1.33 | 13.90 ± 1.29 | 0.157 |
|  | HbA_1c_ (%) | 5.80 ± 0.77 | 5.74 ± 0.69 | 0.084 |
|  | HDL-C (mg/dL) | 61.03 ± 15.90 | 61.66 ± 16.56 | 0.039 |
|  | LDL-C (mg/dL) | 154.69 ± 31.25 | 162.58 ± 42.30 | 0.212 |
|  | Triglycerides (mg/dL) | 134.61 ± 89.25 | 136.18 ± 96.00 | 0.017 |
| **Risk of ASCVD**** | |  |  |  |
| Low | | 497 (6.0) | 209 (8.6) | 0.262 |
| Medium | | 5,539 (66.4) | 1,691 (69.5) |  |
| High | | 1,381 (16.6) | 425 (17.5) |  |
| ≥80 years | | 926 (11.1) | 109 (4.5) |  |
|  | **Medication** |  |  |  |
|  | Statin | 7370 (88.3) | 2155 (88.5) | 0.005 |
|  | Fibrate | 487 (5.8) | 132 (5.4) | 0.018 |
|  | Ezetimibe | 253 (3.0) | 82 (3.4) | 0.019 |

The data are presented as the number (percentage) or as the mean ± standard deviation as appropriate. *Calculated as kg/m^2^. **Risk based on the Hisayama score in patients younger than 80 years. ALT, alanine aminotransferase; ASCVD, atherosclerotic cardiovascular disease; AST, aspartate aminotransferase; BP, blood pressure; GTP, glutamyl transpeptidase; HDL-C, high-density lipoprotein cholesterol; LDL-C, low-density lipoprotein cholesterol; PSM, propensity score matching; SMD, standardized mean difference

**Supplementary Table S6.** Baseline characteristics after propensity score matching

|  | Variable | After PSM | | SMD |
| --- | --- | --- | --- | --- |
|  |  | Frequency of medical visits  in 2 years | |  |
|  |  |  |  |  |
|  |  | ≥22 times | 8–12 times |  |
|  |  | (n=4,812) | (n=1,604) |  |
|  | **Female sex** | 3,228 (67.1) | 1,050 (65.5) | 0.034 |
|  | Mean age (years) | 67.74 ± 6.98 | 67.72 ± 6.96 | 0.003 |
|  | Age group (years) |  |  |  |
|  | 40–49 | 81 (1.7) | 27 (1.7) | <0.001 |
|  | 50–59 | 372 (7.7) | 124 (7.7) |  |
|  | 60–69 | 2,511 (52.2) | 837 (52.2) |  |
|  | 70–79 | 1,644 (34.2) | 548 (34.2) |  |
|  | ≥80 | 204 (4.2) | 68 (4.2) |  |
|  | **Comorbidities** |  |  |  |
|  | Hypertension | 2,131 (44.3) | 705 (44.0) | 0.007 |
|  | Diabetes mellitus | 176 (3.7) | 57 (3.6) | 0.006 |
|  | Cardiac arrhythmias | 321 (6.7) | 106 (6.6) | 0.003 |
|  | Valvular disease | 78 (1.6) | 24 (1.5) | 0.010 |
|  | Renal disease | 88 (1.8) | 36 (2.2) | 0.029 |
|  | Liver disease | 530 (11.0) | 185 (11.5) | 0.016 |
|  | Peptic ulcer | 514 (10.7) | 168 (10.5) | 0.007 |
|  | Rheumatic disease | 125 (2.6) | 42 (2.6) | 0.001 |
|  | Hypothyroidism | 84 (1.7) | 31 (1.9) | 0.014 |
|  | Dementia | 27 (0.6) | 14 (0.9) | 0.037 |
|  | Depression | 191 (4.0) | 65 (4.1) | 0.004 |
|  | Psychoses | 48 (1.0) | 17 (1.1) | 0.004 |
|  | Other neurological disorder | 83 (1.7) | 30 (1.9) | 0.011 |
|  | Iron/B12 deficiency anemia | 119 (2.5) | 40 (2.5) | 0.001 |
|  | **Medical checkup data** |  |  |  |
|  | Current smoker | 467 (9.7) | 156 (9.7) | 0.001 |
|  | Alcohol consumption |  |  |  |
|  | Daily | 792 (16.5) | 269 (16.8) | 0.015 |
|  | Sometimes | 979 (20.3) | 333 (20.8) |  |
|  | Rarely | 3,041 (63.2) | 1,002 (62.5) |  |
|  | Body mass index* | 22.84 ± 3.18 | 22.94 ± 3.28 | 0.031 |
|  | Systolic BP (mmHg) | 131.23 ± 16.98 | 131.34 ± 17.39 | 0.007 |
|  | AST (U/L) | 23.48 ± 7.88 | 23.47 ± 7.94 | 0.001 |
|  | ALT (U/L) | 20.69 ± 12.98 | 20.86 ± 13.07 | 0.013 |
|  | γ-GTP (U/L) | 32.01 ± 35.46 | 32.39 ± 33.56 | 0.011 |
|  | Uric acid (mg/dL) | 5.28 ± 1.30 | 5.31 ± 1.30 | 0.023 |
|  | Creatinine (mg/dL) | 0.74 ± 0.20 | 0.75 ± 0.19 | 0.023 |
|  | Hemoglobin (g/dL) | 13.82 ± 1.30 | 13.90 ± 1.30 | 0.063 |
|  | HbA_1c_ % | 5.75 ± 0.68 | 5.76 ± 0.76 | 0.017 |
|  | HDL-C (mg/dL) | 61.82 ± 16.05 | 61.31 ± 16.18 | 0.032 |
|  | LDL-C (mg/dL) | 159.08 ± 30.15 | 158.62 ± 30.34 | 0.015 |
|  | Triglycerides (mg/dL) | 135.81 ± 92.02 | 135.29 ± 87.49 | 0.006 |
| **Risk of ASCVD**** | |  |  |  |
| Low | | 343 (7.1) | 110 (6.9) | 0.024 |
| Medium | | 3,413 (70.9) | 1,128 (70.3) |  |
| High | | 852 (17.7) | 298 (18.6) |  |
| ≥80 years | | 204 (4.2) | 68 (4.2) |  |
|  | **Medication** |  |  |  |
|  | Statin | 4,292 (89.2) | 1,423 (88.7) | 0.015 |
|  | Fibrate | 265 (5.5) | 95 (5.9) | 0.018 |
|  | Ezetimibe | 137 (2.8) | 45 (2.8) | 0.003 |

The data are presented as the number (percentage) or as the mean ± standard deviation as appropriate. *Calculated as kg/m^2^. **Risk based on the Hisayama score in patients younger than 80 years. ALT, alanine aminotransferase; ASCVD, atherosclerotic cardiovascular disease; AST, aspartate aminotransferase; BP, blood pressure; GTP, glutamyl transpeptidase; HDL-C, high-density lipoprotein cholesterol; LDL-C, low-density lipoprotein cholesterol; PSM, propensity score matching; SMD, standardized mean difference.

**Supplementary Table S7.** Comparison of the 5-year cumulative incidence of the composite outcome, cardiac events, cerebrovascular events, and mortality between the low-frequency visit group (4–6 times in the second year, n=1,894) and the high-frequency visit group (≥11 times in the second year, n=5,833)

| **Outcome** | **Medical visits in year 2, n** | **Events (%)** | **Cumulative incidence at 5 years** | | **p-value** |
| --- | --- | --- | --- | --- | --- |
|  |  |  | **Rate** | **95% CI** |  |
| Composite of events**^†^** | 4–6 | 117 (6.2) | 6.1 | 4.9–7.5 | 0.690 |
|  | ≥11 | 392 (6.7) | 5.3 | 4.7–6.0 |  |
| Cardiac events* | 4–6 | 71 (3.7) | 4.0 | 3.0–5.1 | 0.815 |
|  | ≥11 | 236 (4.0) | 3.2 | 2.7–3.8 |  |
| Cerebrovascular events* | 4–6 | 27 (1.4) | 1.3 | 0.8–2.0 | 0.762 |
|  | ≥11 | 92 (1.6) | 1.3 | 1.0–1.7 |  |
| Death**^†^** | 4-6 | 43 (2.3) | 1.7 | 1.1–2.5 | 0.785 |
|  | ≥11 | 145 (2.5) | 1.8 | 1.4–2.2 |  |

The table includes event counts, percentages, 5-year cumulative incidence, 95% CIs, and p-values. *Gray’s test. ^†^Log-rank test. CI, confidence interval

**Supplementary Table S8.** Comparison of outcomes between the redefined low-frequency visit group (8–16 visits in 2 years, n=1,923) and the high-frequency visit group (≥22 visits in 2 years, 5,924).

| **Outcome** | **Medical visits in 2 years, n** | **Events (%)** | **Cumulative incidence at 5 years** | | **p-value** |
| --- | --- | --- | --- | --- | --- |
|  |  |  | **Rate** | **95% CI** |  |
| Composite of events**^†^** | 8–16 | 144 (7.5) | 6.8 | 5.6–8.2 | 0.854 |
|  | ≥22 | 424 (7.2) | 5.8 | 5.1–6.5 |  |
| Cardiac events* | 8–16 | 87 (4.5) | 4.1 | 3.2–5.2 | 0.955 |
|  | ≥22 | 262 (4.4) | 3.6 | 3.1–4.2 |  |
| Cerebrovascular events* | 8–16 | 36 (1.9) | 1.9 | 1.3–2.7 | 0.574 |
|  | ≥22 | 97 (1.6) | 1.5 | 1.1–1.8 |  |
| Death**^†^** | 8–16 | 53 (2.8) | 1.9 | 1.3–2.8 | 0.956 |
|  | ≥22 | 158 (2.7) | 2.0 | 1.6–2.4 |  |

^*^Gray’s test was performed. ^†^The log-rank test was performed. This table compares the outcomes of cardiac events, cerebrovascular events, and death between the redefined low-frequency visit group (8-16 times/2 years) and the high-frequency visit group (≥22 times/2 years). It includes event counts, percentages, 5-year cumulative incidence, 95% confidence intervals, and p-values.

**Supplementary Table S9.** Comparison of outcomes between the low-frequency visit group (8–12 visits in 2 years, n=1,648) and the intermediate-frequency visit group (13-21 visits in 2 years, 4,758).

| **Outcome** | **Medical visits in 2 years, n** | **Events (%)** | **Cumulative incidence at 5 years** | | **p-value** |
| --- | --- | --- | --- | --- | --- |
|  |  |  | **Rate** | **95% CI** |  |
| Composite of events**^†^** | 8–12 | 88 (5.3) | 5.7 | 4.5–7.1 | 0.669 |
|  | 13–21 | 247 (5.2) | 4.4 | 3.7–5.1 |  |
| Cardiac events* | 8–12 | 58 (3.5) | 3.9 | 2.9–5.1 | 0.380 |
|  | 13–21 | 150 (3.2) | 2.7 | 2.2–3.2 |  |
| Cerebrovascular events* | 8–12 | 20 (1.2) | 1.2 | 0.7–1.9 | 0.830 |
|  | 13–21 | 62 (1.3) | 1.0 | 0.7–1.4 |  |
| Death**^†^** | 8–12 | 27 (1.6) | 1.4 | 0.8–2.2 | 0.520 |
|  | 13–21 | 69 (1.5) | 1.2 | 0.8–1.6 |  |

^*^Gray’s test was performed. ^†^The log-rank test was performed. This table compares the outcomes of cardiac events, cerebrovascular events, and death between the low-frequency visit group (8-12 times/2 years) and the intermediate-frequency visit group (13-21 times/2 years). It includes event counts, percentages, 5-year cumulative incidence, 95% confidence intervals, and p-values.

**Supplementary Table S10.** Comparison of outcomes between the intermediate-frequency visit group (13–21 visits in 2 years, n=4,934) and the high-frequency visit group (≥22 visits in 2 years, 5,244).

| **Outcome** | **Medical visits in 2 years, n** | **Events (%)** | **Cumulative incidence at 5 years** | | **p-value** |
| --- | --- | --- | --- | --- | --- |
|  |  |  | **Rate** | **95% CI** |  |
| Composite of events**^†^** | 13–21 | 320 (6.5) | 5.3 | 4.6–6.0 | 0.897 |
|  | ≥22 | 347 (6.6) | 5.3 | 4.7–6.1 |  |
| Cardiac events* | 13–21 | 193 (3.9) | 3.3 | 2.7–3.9 | 0.669 |
|  | ≥22 | 216 (4.1) | 3.4 | 2.8–4.0 |  |
| Cerebrovascular events* | 13–21 | 71 (1.4) | 1.2 | 0.9–1.6 | 0.492 |
|  | ≥22 | 74 (1.5) | 1.2 | 0.9–1.5 |  |
| Death**^†^** | 13–21 | 97 (2.0) | 1.3 | 1.0–1.8 | 0.125 |
|  | ≥22 | 128 (2.4) | 1.8 | 1.4–2.2 |  |

^*^Gray’s test was performed. ^†^The log-rank test was performed. This table compares the outcomes of cardiac events, cerebrovascular events, and death between the intermediate-frequency visit group (13-21 times/2 years) and the high-frequency visit group (≥22 times/2 years). It includes event counts, percentages, 5-year cumulative incidence, 95% confidence intervals, and p-values.
